# Fatigue Damage Characterisation of Notched Fe-SMA by Weak Magnetic Signals

**DOI:** 10.3390/ma19112215

**Published:** 2026-05-25

**Authors:** Zhi-Yu Xie, Xiang Zhang, Xu Chen, Xi Wu, Shi-Min Zhang

**Affiliations:** 1 School of Civil Engineering and Architecture, Zhejiang University of Science and Technology, Hangzhou 310023, China; 2 Institute of Structural Engineering, College of Civil Engineering and Architecture, Zhejiang University, Hangzhou 310058, China; 3 China Merchants Property Development (Hangzhou) Co., Ltd., Hangzhou 311000, China; 4 Department of Civil Engineering, Hangzhou City University, Hangzhou 310015, China; 5 Zhejiang Engineering Research Center of Intelligent Urban Infrastructure, Hangzhou City University, Hangzhou 310015, China

**Keywords:** iron-based shape memory alloy, notched specimen, weak magnetic effect, fatigue crack, damage localization

## Abstract

**Highlights:**

- Static weak magnetic signals agree well with deformation evolution.
- Fatigue magnetic signals show a clear three-stage evolution.
- Magnetic hysteresis loops reveal richer damage information than deformation loops.
- Post-fracture magnetic anomalies coincide with the crack location.
- Weak magnetic signals can indicate local fatigue damage in Fe-SMA.
- The normal component is more suitable for damage localization and evaluation.
- Combined magnetic monitoring and scanning enable time-space damage assessment.

**Abstract:**

Iron-based shape memory alloys (Fe-SMAs) have considerable potential for the active strengthening of concrete structures, yet convenient externally applicable non-destructive methods for identifying local fatigue damage under cyclic loading remain limited. To investigate the weak magnetic response of notched Fe-SMA and its correspondence with local damage evolution, static tensile tests and constant-amplitude fatigue tests were conducted on Fe-SMA specimens with a semi-circular notch. Weak magnetic signals were continuously monitored at a fixed point throughout loading, and surface magnetic-field scanning was performed after fracture. Under static loading, the magnetic signal evolved consistently with the deformation response. Under fatigue loading, the fixed-point magnetic signal exhibited a clear three-stage evolution corresponding to the development of residual deformation. Compared with deformation hysteresis loops, magnetic hysteresis loops contained richer information on local damage evolution. After fracture, abrupt changes in the scanned magnetic field coincided with the actual fracture location, and the magnetic anomaly gradually attenuated as the scanning path moved away from the notch. These results indicate that weak magnetic signals can effectively characterise the evolution of local fatigue damage in notched Fe-SMA, with the normal magnetic component showing greater sensitivity to damage localisation and state assessment.

## 1. Introduction

Existing concrete structures are commonly exposed to long-term traffic loading, wind loading, temperature variations and other repeated actions during service. Under these conditions, crack propagation, stiffness deterioration and fatigue damage accumulation may gradually develop, thereby impairing structural durability, serviceability and safety [1,2,3,4,5]. Conventional post-repair measures alone are often insufficient to effectively restrain subsequent crack growth [6,7]. Consequently, strengthening techniques capable of providing active intervention have become an important research focus in structural rehabilitation and maintenance, particularly for improving crack control and retarding fatigue damage evolution [8,9].

Iron-based shape memory alloys (Fe-SMAs) have attracted considerable attention in the strengthening of concrete structures owing to their ability to generate recovery stress after pre-straining and thermal activation, thus introducing active prestress into the substrate under restrained conditions [10,11,12,13,14]. Compared with conventional mechanically tensioned prestressing systems, Fe-SMAs do not require complicated jacking equipment, offer greater construction adaptability, and can partially compensate for prestress loss through re-activation. Promising applications have therefore been reported in near-surface mounted (NSM) strengthening, shotcrete overlays, bridge deck strengthening and bridge rehabilitation [15,16,17]. Under repeated loading, Fe-SMA prestressing has also been shown to improve crack control and enhance the fatigue performance of structural members, making it a promising functional material that combines smart activation with structural strengthening [18,19,20].

Despite the advantages mentioned above, Fe-SMA used as a strengthening layer or prestressing element is itself susceptible to fatigue damage under cyclic loading [21,22]. This issue becomes more critical when initial cracks or defects exist on the specimen surface, since stress concentration accelerates crack propagation and promotes more rapid fatigue damage development [23,24,25], which may ultimately compromise the fatigue performance of the strengthened structure. For Fe-SMAs embedded in mortar, concrete or protective layers, local stress concentration, interfacial slip, and the initiation and propagation of fatigue cracks generally occur in regions that are not directly visible. In recent years, distributed optical fibre sensing, digital image correlation (DIC), and piezoelectric sensing have been employed for fatigue crack monitoring or for measuring the local response of Fe-SMA-strengthened systems [26,27,28]. Nevertheless, these methods usually rely on pre-embedded sensors, visual access or sufficient accessible surface area for sensor installation, and therefore remain constrained in embedded Fe-SMA strengthening applications. Moreover, fatigue damage develops progressively over a long period and is usually characterised by weak early-stage manifestations [29,30], making it difficult for conventional strain, displacement or apparent crack observations to capture the internal evolution of fatigue cracks in a timely and continuous manner. The development of a non-destructive approach capable of characterising the local damage state of Fe-SMA from outside the structure, without the need for embedded sensing units, is therefore of clear engineering significance.

In iron-based alloy, the coupling between mechanical and magnetic energies under external stress can induce stress-related spontaneous weak magnetisation, commonly referred to as the weak magnetic effect [31,32,33]. Previous studies have demonstrated that such weak magnetic signals are highly sensitive to the initiation, propagation and instability of fatigue cracks in steel, and can reflect fatigue damage evolution through variations in magnetic field intensity, gradient and spatial distribution characteristics [34,35,36]. More recent studies have further verified the feasibility of weak magnetic or piezomagnetic signals for fatigue damage identification in steel plates, bridge steels, reinforced concrete members and new-to-old concrete interfaces, and have shown that these signals respond well to crack propagation stages and the spatial distribution of damage [37,38,39,40]. By contrast, studies on Fe-SMA remain rather limited, and the relationship between crack development, weak magnetic signals and spatial magnetic field distribution in Fe-SMA under local stress concentration has not yet been clarified. It is therefore necessary to investigate the correlation between the spontaneous weak magnetic signals generated during fatigue crack propagation in Fe-SMA and the corresponding fatigue damage, with a view to extending the weak magnetic effect to the non-destructive detection and monitoring of fatigue cracks.

Against this background, artificially notched Fe-SMA specimens were designed in the present study. Static tensile tests and cyclic tensile fatigue tests were carried out in combination with continuous fixed-point monitoring of weak magnetic signals and post-fracture surface magnetic field scanning, in order to investigate the magnetic response characteristics of Fe-SMA during local crack development. Particular attention was given to the correspondence between local magnetic signals near the notch and deformation evolution under static and cyclic loading, as well as to the relationship between post-fracture magnetic anomalies, crack location and their spatial distribution characteristics. The study is expected to provide experimental evidence for the non-destructive identification of local fatigue cracks in Fe-SMA and to offer a reference for the future development of service-state monitoring methods for embedded Fe-SMAs in concrete structures.

## 2. Materials and Methods

### 2.1. Material and Specimen Preparation

**Figure 1:**
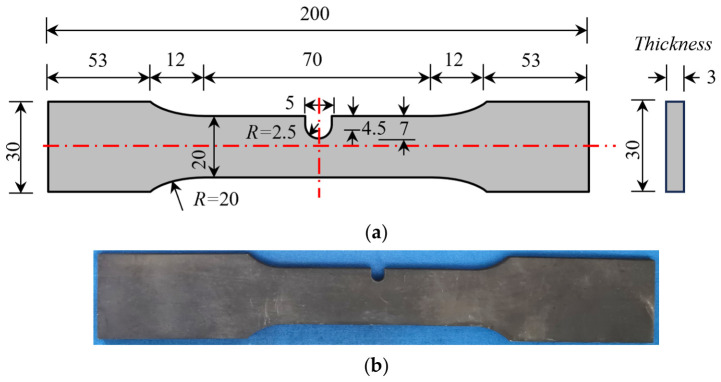
Specimen design and photographs: (**a**) specimen design (unit: mm); (**b**) photograph of specimen.

Fe-SMA alloy specimens were used in this study. The material was supplied by Zhongsheng Xulong Co., Ltd., Dalian, China, with a nominal chemical composition of Fe-17Mn-5Si-10Cr-5Ni (wt.%) (as shown in Table 1). The alloy was produced by melting in a medium-frequency vacuum induction furnace under a vacuum level of 1.33 Pa (approximately $10^{-2}$ Torr). After complete melting of the raw materials, the melt was held for 30 min to improve chemical homogeneity and was subsequently cast into metallic moulds to form ingots. The ingots were hot-rolled at 900–1200 °C into plates with a thickness of 3 mm, followed by machining into the required test specimens. The test specimen is shown in Figure 1. Dog-bone specimens were prepared in accordance with GB/T 228.1-2021 [41]. In addition, a semi-circular notch was introduced at the middle of one side of the specimen to generate a stable local stress concentration zone during loading and to ensure a well-defined location for crack initiation and propagation. The specimens had an overall length of 200 mm, a thickness of 3 mm, a reduced-section width of 20 mm, a notch radius of 2.5 mm and a notch depth of 4.5 mm.

### 2.2. Test Setup

To investigate the mechanical response and magnetic field evolution of notched Fe-SMA specimens under monotonic and cyclic tensile loading, both static and fatigue tests were carried out, as summarised in Table 2. Two parallel specimens were prepared for each test series, denoted as STC and FAC for the static and fatigue groups, respectively. Based on the ultimate strength obtained from the static tests, the maximum stress level adopted in the fatigue tests was set to 60% of the ultimate strength, while the minimum stress level was set to 6% of the ultimate strength, corresponding to a stress ratio of 0.1. The static tests were conducted to determine the ultimate load-carrying capacity and fracture deformation characteristics of the notched specimens, and also to provide a basis for selecting the fatigue loading parameters. The fatigue tests were then performed to track the development of local damage near the notch under cyclic loading together with the corresponding magnetic field response.

**Figure 2:**
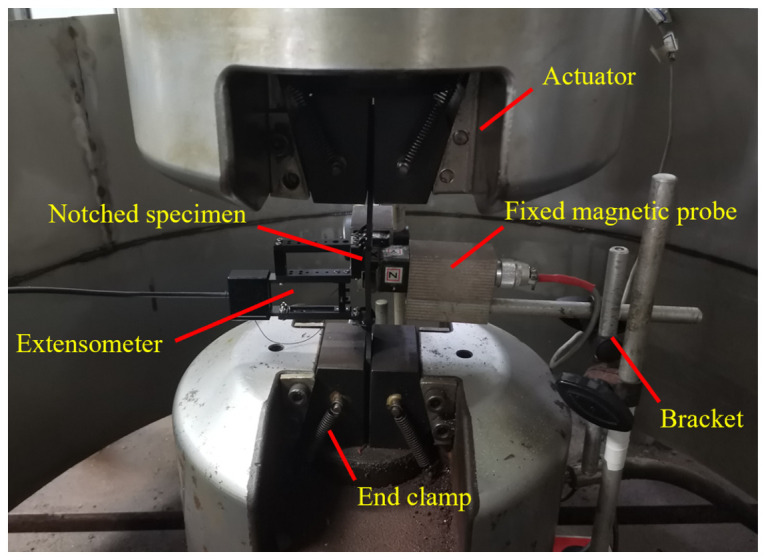
Loading and test setup.

**Figure 3:**
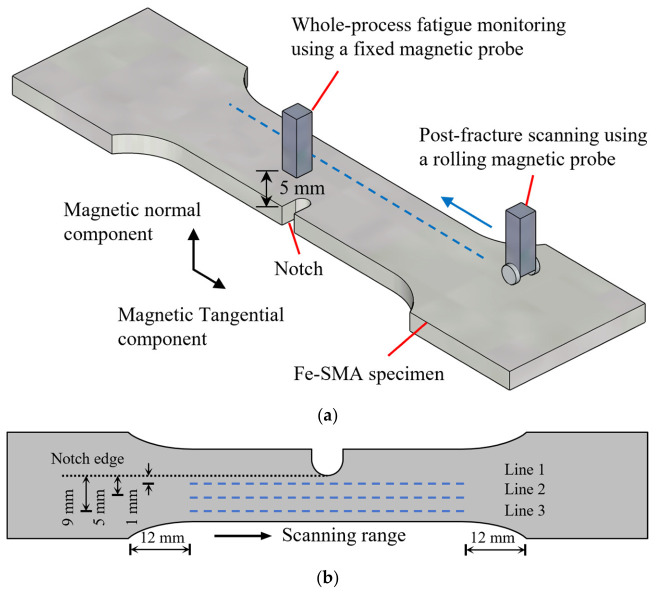
Magnetic field measurement scheme: (**a**) magnetic measurement arrangement; (**b**) magnetic field scanning line arrangement.

The loading and measurement arrangement is shown in Figure 2 and Figure 3. Both the static and fatigue tests were conducted using a 25-ton electro-hydraulic servo fatigue testing machine (SDS-250, Sinotest Equipment Co., Ltd., Changchun, China). The specimens were clamped at both ends, with a gripping length of 45 mm. The applied load was recorded by the built-in load cell, while the deformation at the middle of the specimen was measured by the extensometer integrated into the testing machine. The static tests were performed under displacement control at a rate of 0.5 mm/min in order to obtain sufficiently stable and well-resolved mechanical–magnetic response curves during monotonic tension, while avoiding insufficient data acquisition during the critical stage immediately before fracture caused by excessively rapid loading. The fatigue tests were conducted under constant-amplitude load control at 4 Hz so as to balance testing efficiency while minimising significant temperature rise and additional dynamic effects. To continuously monitor the local magnetic field variation during loading, a fluxgate meter (MODEL-191) from Zhongyuhuantai Co., Ltd., Qingdao, China was fixed near the notch. The probe was positioned above the notch with a lift-off distance of 5 mm from the specimen surface so as to capture the magnetic field evolution within the local stress concentration zone during both static and fatigue loading. The instrument simultaneously recorded the normal and tangential components of magnetic flux density at the measurement point. During the measurements, a permalloy ring was used to reduce interference from the surrounding environmental magnetic field. Meanwhile, surface magnetic field scanning was carried out after fracture in order to characterise the spatial magnetic field distribution around the fractured region. The scanning was performed using a magnetic memory detector (TSC-8M) from Energodiagnostika Co. Ltd., Moscow, Russia. As shown in Figure 3, three parallel scanning paths (shown as the blue dotted lines) were arranged below the notch along the specimen axis, with vertical distances of 1 mm, 5 mm and 9 mm from the pre-fabricated notch, respectively. Both the normal and tangential magnetic-field components were recorded during surface scanning and fixed-point monitoring. The normal component refers to the magnetic-field component perpendicular to the notch, whereas the tangential component refers to that along the specimen length. The field directions are indicated in Figure 3. This arrangement enabled comparison of the amplitude and attenuation characteristics of the post-fracture magnetic anomalies at different distances, thereby allowing the spatial spread of magnetic field redistribution induced by local damage to be analysed. By combining fixed-point magnetic monitoring with post-fracture surface scanning, the magnetic response of notched Fe-SMA specimens could be characterised from both temporal and spatial perspectives.

## 3. Results and Discussion

### 3.1. Static Test Results

**Figure 4:**
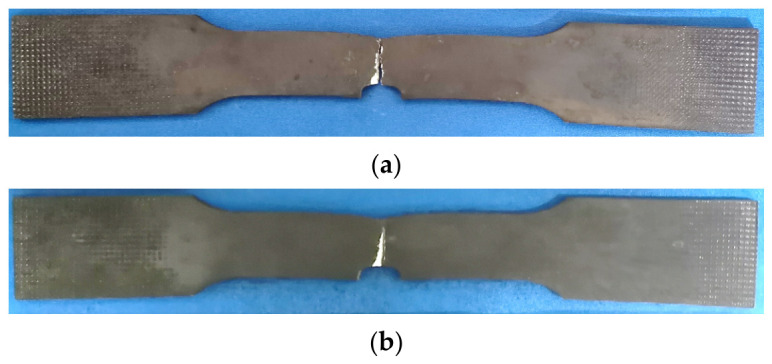
Specimens after failure under static loading: (**a**) STC-1; (**b**) STC-2.

The static tensile test results of the notched Fe-SMA specimens are summarised in Table 3, and the final failure modes are shown in Figure 4. It can be seen that both specimens fractured within the notched region. The ultimate strength values reported in this study were all defined in terms of the nominal stress based on the net section at the notch. The corresponding nominal stress was calculated as follows [42]:

(1) $$A_{net}=t(b-d_{n}),$$

(2) $$\sigma =\frac{F}{A_{net}},$$

where $A_{net}$ is the net cross-sectional area, t is the specimen thickness, b is the width of the reduced section, and $d_{n}$ is the depth of the single-sided notch. The ultimate strengths of the two specimens were 600.0 MPa and 634.4 MPa, respectively, while the corresponding fracture deformations were 11.9 mm and 12.3 mm. Considering the presence of the notch, the strain reported herein refers to the average strain over the selected 70 mm gauge length, calculated as the deformation within the gauge length divided by the initial gauge length. The corresponding values were 17.0% and 17.6%, respectively. These results indicate that the notched Fe-SMA specimens still exhibited relatively high load-carrying capacity and good deformation ability under monotonic tension. The static failure process of the notched specimens was mainly governed by deformation and fracture under local stress concentration.

**Figure 5:**
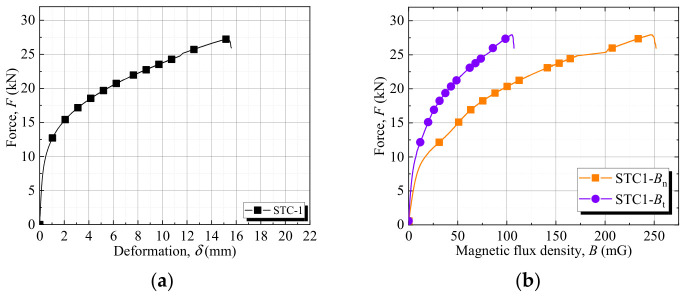

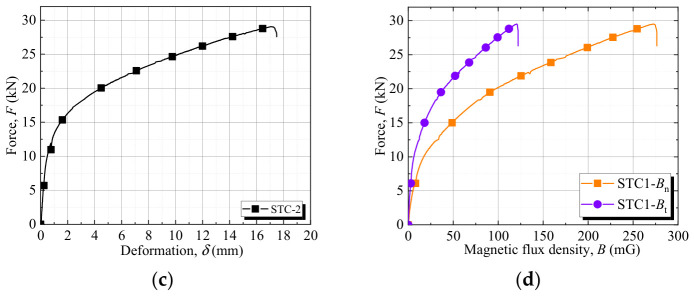
Comparison of force-displacement curve and force-magnetic signal curve under static loading: (**a**) force-deformation of STC-1; (**b**) force-magnetic signal of STC-1; (**c**) force-deformation of STC-2; (**d**) force-magnetic signal of STC-2.

Figure 5 presents the load-displacement curves and load-magnetic signal curves of the two specimens under static loading. For both STC-1 and STC-2, the load-displacement response exhibited a pronounced non-linear evolution. The load increased rapidly at the initial stage and then gradually entered a slow hardening stage with increasing deformation until final fracture occurred in the notched region. Consistent with the mechanical response, the magnetic signals also showed a continuous non-linear evolution during loading, and their overall variation trends agreed well with those of the load–displacement curves. This indicates that the magnetic response of notched Fe-SMA under monotonic loading can effectively follow the evolution of local stress and deformation. Further comparison between the normal and tangential magnetic components shows that the amplitude of the normal magnetic signal was markedly greater than that of the tangential component, and its increase with load was also more pronounced. This suggests that the normal component is more sensitive to the local stress concentration and magnetisation variation near the notch. These observations provide a basis for the subsequent analysis of the magnetic signal under cyclic loading.

### 3.2. Fatigue Test Results

**Figure 6:**
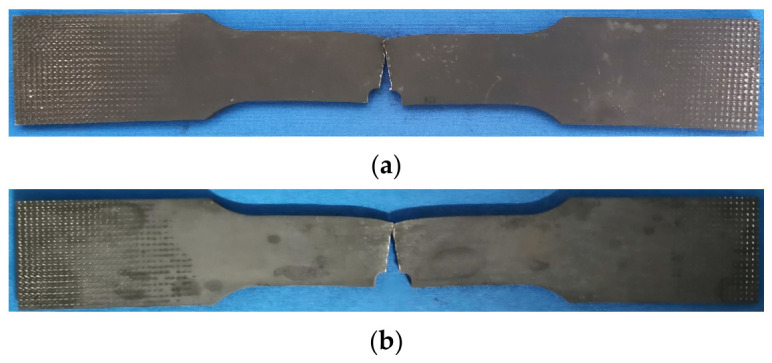
Specimens after failure under cyclic loading: (**a**) FAC-1; (**b**) FAC-2.

Figure 6 shows the final failure modes of the notched Fe-SMA specimens under cyclic loading, and the corresponding fatigue test results are summarised in Table 4. Both specimens fractured in the notched region, and the fracture locations coincided with the pre-defined weak section, indicating that the notch continuously induced local stress concentration under cyclic loading and governed the initiation and propagation path of fatigue cracks. Under the same loading condition, the fatigue lives of FAC-1 and FAC-2 were 13,985 and 16,870 cycles, respectively, while the elongations after fracture were 10.1% and 6.0%, respectively. The relatively limited difference in fatigue life between the two specimens suggests that the notched Fe-SMA specimens exhibited a reasonably stable fatigue response under the present loading condition. At the same time, the difference in post-fracture elongation between FAC-1 and FAC-2 indicates that, although fatigue failure in both cases originated from the notched region, certain specimen-to-specimen differences still existed in the degree of local plastic development during crack propagation and in the deformation accumulation prior to final fracture.

#### 3.2.1. Analysis of Full-Process Magnetic Signal Monitoring

**Figure 7:**
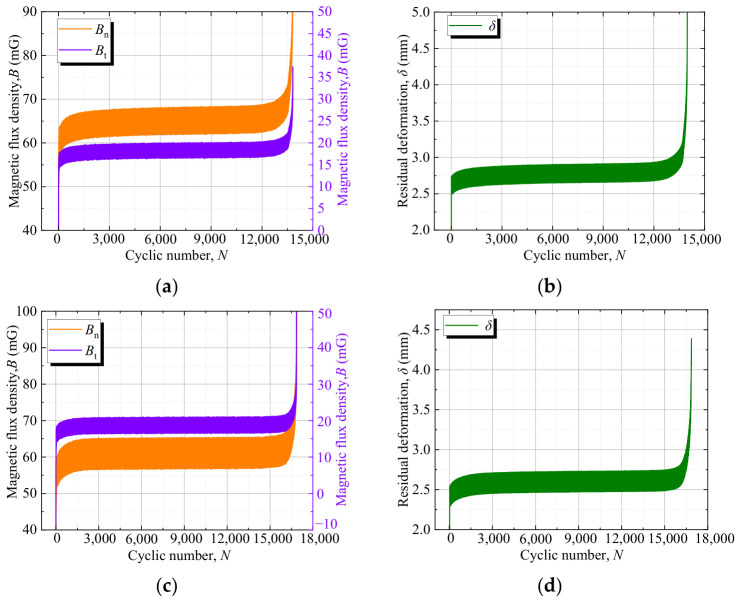
Comparison of Magnetic signal variation and deformation variation under cyclic loading: (**a**) magnetic signal variation in FAC-1; (**b**) deformation variation in FAC-1; (**c**) magnetic signal variation in FAC-2; (**d**) deformation variation in FAC-2.

Figure 7 presents the evolution of the fixed-point normal magnetic signal $B_{n}$, tangential magnetic signal $B_{t}$, and residual deformation δ with the number of cycles N for FAC-1 and FAC-2 under cyclic loading. It can be seen that the residual deformation of both specimens exhibited a typical three-stage evolution, i.e., a rapid increase at the initial fatigue stage, followed by a relatively long stage of slow development, and finally a sharp acceleration prior to fracture. Correspondingly, the magnetic signals recorded at the fixed point also showed clear stage-wise variation, characterised by a rapid adjustment at the initial stage, a gradual evolution in the intermediate stage, and a sudden increase as failure was approached. Further comparison of the two magnetic components shows that the normal magnetic signal $B_{n}$ consistently exhibited a higher response amplitude and clearer stage transitions throughout the entire fatigue process, whereas the tangential magnetic signal $B_{t}$ followed a trend broadly similar to that of $B_{n}$ but with a smaller overall fluctuation amplitude. In particular, the abrupt increase in $B_{n}$ became much more pronounced near failure and corresponded well to the sharp increase in residual deformation. These results indicate that, in notched Fe-SMA specimens, the local magnetic response can effectively track the full evolution of fatigue damage. This can be attributed to the progressive development of plastic deformation and microcracking at the notch root under cyclic loading, which continuously redistributed the local stress field and magnetisation state, while the leakage-field effect near the crack tip and its surrounding region was progressively intensified [35]. As a result, the magnetic signal recorded by the fixed probe exhibited stage-wise evolution consistent with that of the residual deformation. The above observations not only confirm that Fe-SMA exhibits stable and identifiable magnetic response characteristics under cyclic loading, but also demonstrate that the fixed-point magnetic probe can effectively capture the entire process from local plastic development to final unstable fracture in the notched region, thereby providing a basis for continuous monitoring of local fatigue damage in notched members.

**Figure 8:**
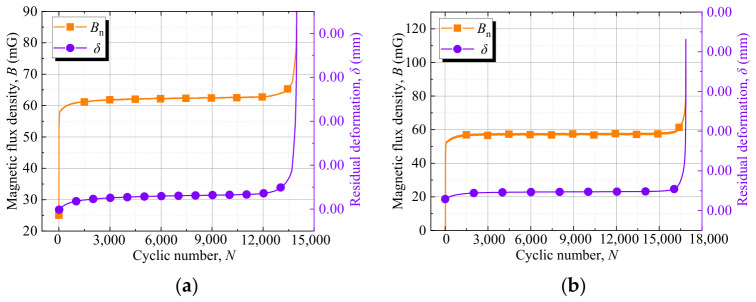
Comparison of magnetic signal and deformation characteristic curves throughout loading: (**a**) FAC-1; (**b**) FAC-2.

Since the normal magnetic signal showed a stronger response and clearer evolution throughout the fatigue process, a further comparison was made between the normal magnetic characteristic parameter (select the residual magnetic signals after each unloading) and the residual deformation δ, as shown in Figure 8. For both FAC-1 and FAC-2, the normal magnetic characteristic parameter maintained good correspondence with the residual deformation over the entire fatigue life: it increased rapidly at the initial stage, accumulated gradually during the intermediate stage, and rose sharply as fracture approached. During the stable development stage, the normal magnetic characteristic parameter continued to evolve, indicating that the internal magnetisation state and local stress state in the notched region did not remain unchanged, but were gradually adjusted as microscopic damage accumulated. In the final stage prior to failure, the normal magnetic characteristic parameter and the residual deformation increased almost synchronously, suggesting that local crack propagation, plastic concentration, and magnetic field redistribution all became markedly intensified during this stage. Compared with the raw magnetic signal, the characteristic parameter reduced the influence of instantaneous intra-cycle fluctuations, making the transitions between different stages clearer and facilitating the identification of key turning points in fatigue damage evolution. Therefore, rather than relying solely on the raw magnetic time-history curves, the magnetic characteristic parameter extracted from the normal component appears more suitable for evaluating the fatigue state of notched Fe-SMA specimens. It provides results that are consistent with the evolution of residual deformation during notch-controlled fatigue failure.

#### 3.2.2. Analysis of Hysteresis Loops During Cyclic Loading

**Figure 9:**
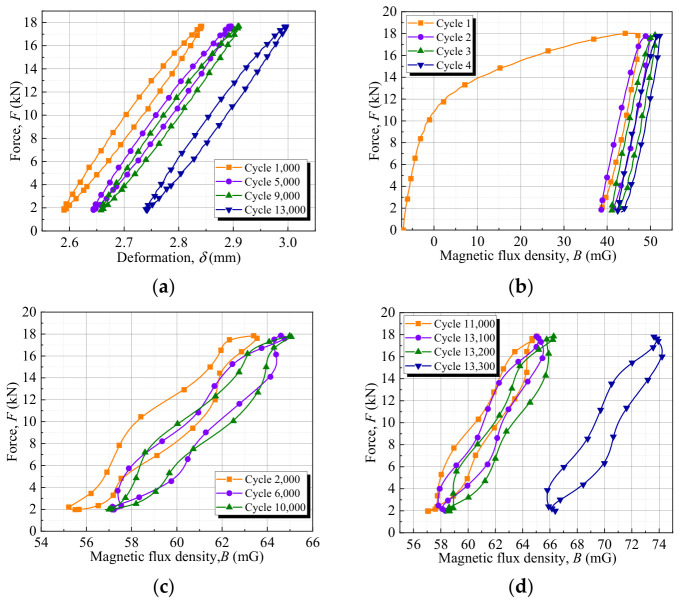
Hysteresis loop curves during loading: (**a**) deformation hysteresis curves; (**b**) magnetic hysteresis curves: initial stage; (**c**) magnetic hysteresis curves: middle stage; (**d**) magnetic hysteresis curves: final stage.

Taking FAC-1 as an example, Figure 9 presents the deformation hysteresis loops and the normal magnetic hysteresis loops at representative cycle numbers under cyclic loading. As shown in Figure 9a, the evolution of the deformation hysteresis loops was relatively simple. The overall curve shape changed only slightly from one cycle number to another, and the main feature was a gradual shift towards larger deformation with increasing cycles, reflecting the continuous accumulation of residual deformation. This indicates that, at the macroscopic mechanical-response level, the fatigue behaviour of the notched specimen was still mainly characterised by gradual stiffness degradation and residual deformation growth, whereas the shape of the loop within an individual cycle did not undergo particularly complex changes.

By contrast, the normal magnetic hysteresis loops exhibited much richer evolutionary features. Figure 9b shows that, at the initial loading stage, the magnetic response in the first cycle differed markedly from that in the subsequent cycles, with both the loop position and shape undergoing obvious adjustment, indicating that the specimen experienced rapid magnetic-state reconstruction during the first loading process. Over the next few cycles, the magnetic hysteresis loops gradually became more concentrated, while still retaining a noticeable residual magnetic response, suggesting that, under the stress concentration near the notch, the local magnetisation state did not undergo a purely reversible change, but was accompanied by a certain degree of irreversible accumulation during loading and unloading [38]. In the intermediate fatigue stage (Figure 9c), the magnetic hysteresis loops not only shifted progressively towards higher magnetic flux density, but also continued to change in shape, evolving from a slender form to a fuller one. This indicates that the local magnetic response near the notch during the intermediate fatigue stage was jointly affected by cumulative local plasticity, the initiation of microcracks, and the increasing non-uniformity of magnetisation [31]. As failure approached (Figure 9d), the magnetic hysteresis loops underwent an obvious overall rightward shift and a marked change in shape. In particular, immediately prior to final fracture, when the macro-crack propagated rapidly through the highly stressed region, the magnetic flux lines near the crack surfaces were strongly disturbed, and the local leakage field was enhanced and redistributed [34,37]. Consequently, the loop increased rapidly, and distortion became evident. Overall, the deformation hysteresis loops of notched Fe-SMA specimens under cyclic loading mainly reflected the accumulation of residual deformation, whereas the normal magnetic hysteresis loops were able to characterise local magnetic-state reconstruction, damage accumulation, and the abnormal changes preceding instability. Compared with conventional deformation hysteresis loops, the magnetic hysteresis loops contained richer information on local damage evolution throughout the entire fatigue process and exhibited higher sensitivity to the transition towards unstable crack propagation.

#### 3.2.3. Analysis of Magnetic Field Distribution Curves

**Figure 10:**
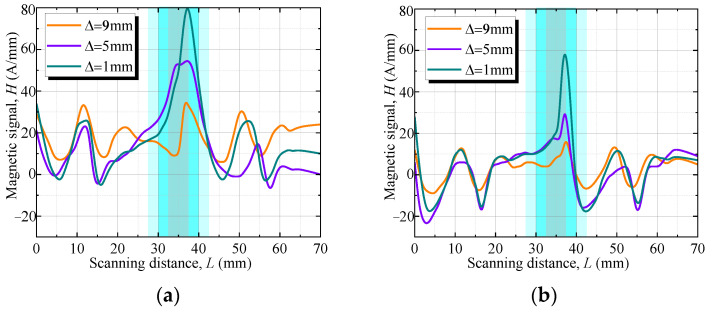

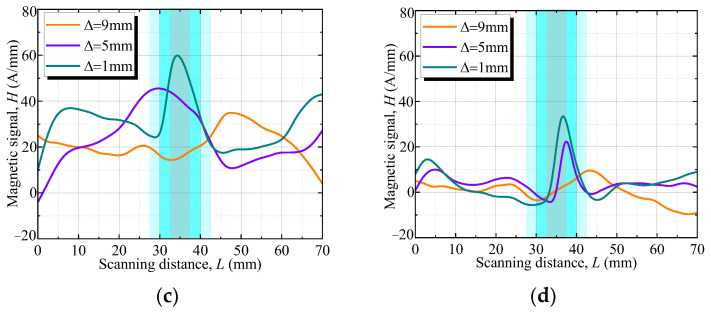
Normal and tangential magnetic field distribution after fracture: (**a**) normal magnetic field distribution FAC-1; (**b**) tangential magnetic field distribution FAC-1; (**c**) normal magnetic field distribution FAC-2; (**d**) tangential magnetic field distribution FAC-2.

Figure 10 presents the surface magnetic field scanning results of the two notched Fe-SMA specimens after fatigue fracture. The three coloured bands represent the notch region, the twofold notch-affected region, and the threefold notch-affected region, respectively. It can be seen that the surface magnetic field distribution changed markedly, with pronounced peaks, abrupt variations and intensified local fluctuations appearing in the vicinity of the fractured region, indicating that fatigue fracture induced distinct local magnetic anomalies near the notch. For both FAC-1 and FAC-2, and for both the normal and tangential magnetic components, the mutation positions in the scanning curves corresponded well to the actual fracture locations, demonstrating that post-fracture surface magnetic field scanning can effectively identify the location of local damage. This is mainly attributed to the redistribution of stress and magnetisation states near the notch after fracture, together with the formation of local leakage fields around the crack surfaces and fractured region, which caused the fractured zone to appear as a distinct anomaly in the scanning curves. Compared with the tangential magnetic field, the normal magnetic field showed a more pronounced response to fracture-induced anomalies. In Figure 10a,c, the normal magnetic field generally exhibited higher peaks and more concentrated local mutations in the fractured region, making the anomalous location clearer. By contrast, although the tangential magnetic field in Figure 10b,d also reflected the post-fracture magnetic field distortion, its anomaly features were more dispersed. This indicates that the local leakage field after fracture gives a stronger response in the surface-normal direction.

In addition, the relative distance between the scanning path and the notch had a significant influence on the amplitude of the magnetic anomaly. For both magnetic components in the two specimens, the strongest local anomaly was observed at Δ=1 mm. As the scanning distance increased to 5 mm and 9 mm, both the peak value and the amplitude of the local fluctuations gradually decreased, and the signal contrast associated with the fractured region became noticeably weaker. This indicates that the fracture-induced magnetic anomaly exhibited clear spatial localisation and distance-dependent attenuation: the closer the scanning path was to the notch region, the more readily the strong local leakage-field response could be captured; as the scanning path moved away from the damage source, the local anomalous field gradually decayed and was smoothed by the background magnetic field. Overall, although some differences existed in the local waveform details between the two specimens, both showed the same general features, namely identifiable fracture location, stronger normal response, and progressive attenuation of anomalies with increasing distance, indicating good repeatability of this behaviour.

To quantitatively characterise the local anomaly features of the post-fracture magnetic field distribution and their variation with scanning distance, two characteristic indicators were defined, namely the magnetic peak value $H_{p}$ and the magnetic anomaly amplitude ΔH. The magnetic peak value $H_{p}$ was defined as the principal anomalous peak in the scanning curve:

(3) $$H_{p}=max[H(x)],$$

where H(x) is the magnetic signal measured along the scanning path, and x is the scanning position coordinate. $H_{p}$ was used to represent the maximum response intensity of the local magnetic anomaly after fracture.

The magnetic anomaly amplitude ΔH was defined in terms of the peak-to-base difference as follows:

(4) $$\Delta H=H_{p}-\frac{H_{L,base}\;+\;H_{R,base}}{2},$$

where $H_{L,base}$ and $H_{R,base}$ are the left and right reference base values of the principal peak, respectively, defined as either the minimum magnetic signal values between the peak position and the corresponding scanning boundaries, or the adjacent valley values on either side of the peak. This parameter reflects the degree of local mutation of the principal anomalous peak relative to the background level on both sides.

**Figure 11:**
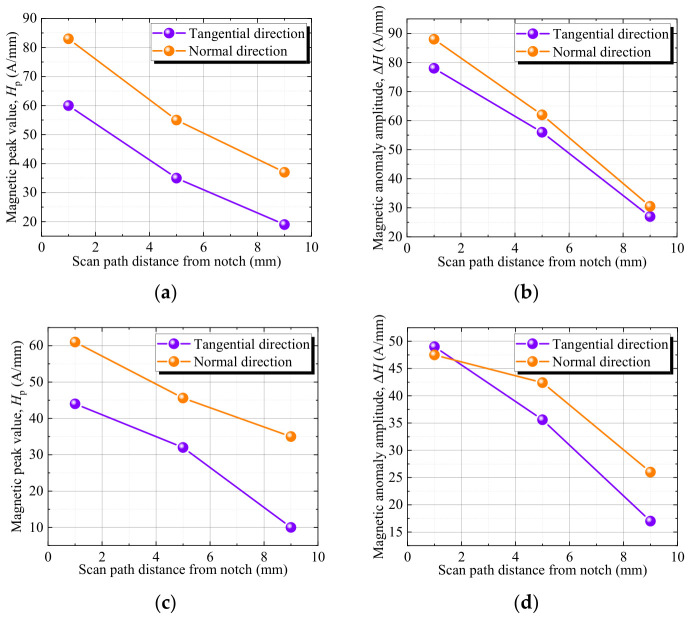
Comparison of characteristic indicators of magnetic field distribution: (**a**) FAC-1; (**b**) FAC-1; (**c**) FAC-2; (**d**) FAC-2.

Figure 11 shows the variation in the magnetic peak value $H_{p}$ and magnetic anomaly amplitude ΔH with the scanning distance from the notch for the two fractured specimens. Regardless of whether $H_{p}$ or ΔH was used, both specimens exhibited the same trend: as the scanning distance increased from Δ=1 mm to 5 mm and 9 mm, both characteristic parameters decreased continuously, indicating that the local magnetic anomaly after fracture gradually weakened with increasing distance from the notch. In terms of the magnetic peak value $H_{p}$, the normal magnetic field was consistently higher than the tangential magnetic field in both specimens, indicating that the normal component responded more strongly to fracture-induced local magnetic field distortion. Along the path closest to the notch, the normal direction exhibited the largest peak values. As the scanning path moved progressively away from the notch, both the normal and tangential peak values decreased, while the normal component remained at a consistently higher level. This suggests that the local leakage field after fracture was more concentrated in the normal direction, and that the normal magnetic field therefore had a stronger capability for indicating the location of local damage. The variation in ΔH was broadly consistent with that of $H_{p}$. For both FAC-1 and FAC-2, ΔH decreased markedly with increasing scanning distance, and the values in the normal direction remained generally higher than those in the tangential direction. This indicates that the post-fracture magnetic anomaly was reflected not only in the increase in the absolute peak value, but also in the enhanced local mutation of the peak relative to the background magnetic field on either side. In other words, the closer the scanning probe was to the notch region, the more readily it captured the strong local leakage-field response jointly induced by the crack surfaces, fracture edges and local stress release. Overall, both $H_{p}$ and ΔH effectively characterised the spatial distribution features of the post-fracture magnetic anomaly. $H_{p}$ directly reflects the maximum response level of the local anomaly, whereas ΔH further describes the degree of local mutation of the anomalous peak relative to the background signal on both sides. Both parameters indicate that the post-fracture magnetic anomaly was mainly concentrated near the notch, and that the normal magnetic field exhibited higher response intensity and stronger characterisation capability for local fracture damage.

From the above analysis, it can be seen that both the normal and tangential magnetic components exhibited sensitivity to the damaged region, and both showed attenuation with increasing scanning distance. Compared with the tangential magnetic field, the normal component displayed higher anomaly intensity and clearer local mutation features. The normal signal is therefore more suitable for identifying and locating damage, and may also offer potential for characterising the extent of damage influence and for delineating damaged zones. This aspect, however, requires further investigation.

### 3.3. Mechanism Analysis of Weak Magnetic Response

**Figure 12:**
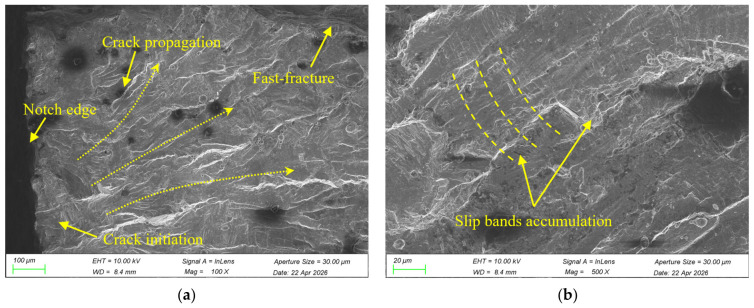
SEM morphology of the fatigue fracture section: (**a**) cross-section near notch edge; (**b**) crack propagation zone.

To further clarify the correspondence between fatigue damage and the weak magnetic response in notched Fe-SMA, the underlying mechanism was discussed in conjunction with the SEM morphology of the fatigue fracture surfaces. As shown in Figure 12, the fatigue fracture surface can be divided into a crack initiation zone, a stable propagation zone, and a final fast-fracture zone. The crack initiation zone was located near the notch root, indicating that local stress concentration at the notch governed crack initiation. The stable propagation zone suggests that the crack underwent a prolonged stage of gradual growth under cyclic loading, whereas the final fast-fracture zone corresponds to rapid unstable fracture after the remaining effective cross-section had been significantly weakened. This fracture zonation agrees well with the three-stage evolution observed in the magnetic signals and residual deformation. Specifically, the early stage mainly corresponds to the rapid reconstruction of the local magnetisation state at the notch root under stress concentration; the intermediate stage corresponds to continuous damage accumulation and stable crack propagation, during which the local magnetic state evolves gradually with crack growth and the development of the plastic zone; and the final stage corresponds to rapid crack propagation and unstable fracture immediately prior to failure, accompanied by a marked enhancement of the magnetic anomaly. As shown in the preceding sections, both fatigue specimens fractured in the notched region; both the fixed-point magnetic signals and the residual deformation exhibited a clear three-stage evolution; and the abrupt changes in the post-fracture scanned magnetic field coincided well with the actual fracture location.

**Figure 13:**
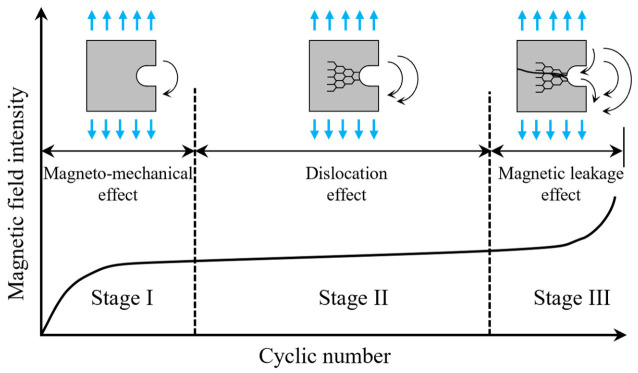
Schematic illustration of the weak magnetic response mechanism in notched Fe-SMA during fatigue loading.

From a mechanistic perspective, the weak magnetic response of notched Fe-SMA during fatigue is mainly associated with the combined effects of the magneto-mechanical effect, the evolution of dislocations and defects, and magnetic flux leakage from the crack surfaces, as illustrated in Figure 13. First, under cyclic tension, stress concentration in the notched region induces local reorientation of magnetic domains and changes in the magnetisation state, thereby giving rise to an overall weak magnetic response at the macroscopic level [31,38]. Secondly, as the number of cycles increases, local plastic deformation accumulates continuously at the notch root, while dislocations, slip bands, and microcracks progressively develop. Through the pinning effect, these microstructural defects hinder domain-wall motion, resulting in continuous reconstruction of the local magnetic state and, consequently, stage-wise changes in the magnetic hysteresis loops [37]. Finally, once the crack enters the final instability stage, the local magnetic flux leakage near the crack surfaces increases sharply. As a result, the monitored magnetic signal shows an abrupt change in the late fatigue stage, and the post-fracture scanning curves exhibit pronounced peaks and abrupt variations in the fractured region. This phenomenon is consistent with previous studies, which have shown that, during fatigue crack propagation in steel, weak magnetic signals continue to evolve with crack growth and are significantly correlated with crack length, stress state, and fatigue damage [34]. The present results further indicate that this approach is also applicable to the identification of local fatigue damage in notched Fe-SMA.

In summary, the weak magnetic response of notched Fe-SMA can be understood as the combined result of magnetisation-state adjustment induced by local stress concentration, continuous magnetic-state reconstruction caused by cyclic plasticity and microdefect accumulation, and enhanced magnetic flux leakage associated with crack propagation. Among the measured components, the normal magnetic component showed greater sensitivity to damage evolution and fracture location, which also provides a mechanistic explanation for the three-stage magnetic-signal evolution, the changes in the magnetic hysteresis loops, and the distribution of post-fracture magnetic anomalies observed in this study. Owing to space limitations, the quantitative correlation among fatigue damage state, stress concentration factor, and magnetic signal is not presented here; this topic is currently under further investigation.

## 4. Conclusions

To investigate the weak magnetic response of notched iron-based shape memory alloy Fe-SMA under static and cyclic tension, this study carried out static and fatigue tests on notched Fe-SMA specimens, during which fixed-point magnetic signal monitoring and post-fracture surface magnetic field scanning were performed. Based on the experimental results, the following conclusions can be drawn:

1. Notched Fe-SMA specimens exhibited pronounced local fracture-controlled behaviour under static loading. The load-displacement curves showed clear non-linear evolution, and the load-magnetic signal curves followed a generally similar trend, indicating that the magnetic response under static loading can effectively track the development of local deformation. Compared with the tangential component, the normal magnetic signal showed a larger amplitude and higher sensitivity to local stress concentration and magnetisation variation near the notch.

2. Under cyclic loading, the magnetic signals obtained from fixed-point monitoring were able to characterise the full evolution of local fatigue damage near the notch and exhibited a typical three-stage evolution. The extracted characteristic parameter reduced the influence of instantaneous intra-cycle fluctuations, making the different fatigue stages and their transition points clearer. The normal magnetic signal showed a stronger response and a more pronounced increase prior to failure. These results indicate that the local magnetic response near the notch can effectively track the damage evolution process from plastic accumulation and microcrack development to final unstable fracture.

3. Compared with the deformation hysteresis loops, the magnetic hysteresis loops contained richer information on local damage evolution, showing clear overall migration and progressive degradation of loop morphology at different fatigue stages. The magnetic hysteresis loops were therefore more sensitive to the instability process of local damage.

4. For both fatigued specimens, the post-fracture surface magnetic field distribution exhibited distinct peaks, abrupt variations, and intensified local fluctuations near the fractured region, and the mutation positions in the scanning curves corresponded well to the actual fracture locations. The normal magnetic signal was more pronounced than the tangential one. As the distance between the scanning path and the notch increased, the magnetic signals in both components decreased continuously, indicating that the fracture-induced magnetic anomaly exhibited pronounced spatial localisation and distance-dependent attenuation.

5. Two indicators, namely the principal peak value and the magnetic anomaly amplitude, were defined to characterise the fluctuation level of the magnetic field distribution. The results show that both the normal and tangential magnetic components were sensitive to the damaged region and exhibited clear attenuation with increasing scanning distance. Compared with the tangential component, the normal magnetic field showed higher anomaly intensity and clearer local mutation features, indicating greater potential for damage identification and localisation. It may also be useful for characterising the extent of damage influence and for delineating damaged zones, although this requires further study.

6. SEM observations of the fatigue fracture surfaces showed that the fracture of notched Fe-SMA could be divided into a crack initiation zone, a stable propagation zone, and a final fast-fracture zone, with crack initiation occurring at the notch root, indicating that local stress concentration governed the initiation and propagation of fatigue cracks. This fracture zonation agrees well with the three-stage evolution of the weak magnetic signals and residual deformation, suggesting that the weak magnetic response of notched Fe-SMA mainly arises from magnetisation-state adjustment induced by local stress concentration, continuous reconstruction of the magnetic state caused by cyclic plasticity and microdefect accumulation, and the enhancement of magnetic flux leakage during the rapid crack propagation and instability stage.

The present study still has certain limitations. First, the number of specimens remains limited. As the main focus of this work was to verify the identifiability and repeatability of the weak magnetic response to fatigue damage in notched Fe-SMA, no sufficiently robust conclusions can yet be drawn regarding the statistical distribution of fatigue life or magnetic characteristic parameters. Secondly, during loading, the current magnetic measurement approach mainly provides time-history responses at a fixed point and is therefore unable to characterise in real time the spatial distribution of the magnetic field around the notch. Moreover, although magnetic shielding was adopted under laboratory conditions to reduce interference from the environmental magnetic field, effective shielding and background-field compensation in complex engineering magnetic environments still require further study. Future work will therefore involve increasing the sample size, extending the range of stress levels and notch parameters, and combining notch geometry, local stress-field analysis, and multi-point magnetic measurements to investigate further the statistical evolution characteristics of magnetic signals and their quantitative relationship with the degree of local stress concentration.

## Figures and Tables

**Table 1:** Nominal chemical composition of Fe-SMA (wt.%).

Fe	Mn	Si	Cr	Ni
balance	17	5	10	5

**Table 2:** Test specimens and experiment design.

Test Group	Specimen Notation	Load Method	*S*_max_	*S*_min_
1	STC-1	Static	100%	\
	STC-2	Static	100%	\
2	FAC-1	Fatigue	60%	6%
	FAC-2	Fatigue	60%	6%

Note: *S*_max_ denotes the ratio of the maximum applied stress to the ultimate tensile strength, and *S*_min_ denotes the ratio of the minimum applied stress to the ultimate tensile strength.

**Table 3:** Static test results.

No.	Specimen Notation	Ultimate Strength *σ*_u_ [MPa]	Deformation *δ* [mm]	Strain *ε* [%]
1	STC-1	600.0	11.9	17.0
2	STC-2	634.4	12.3	17.6

**Table 4:** Fatigue test results.

No.	Specimen Notation	Mean Stress *σ*_m_/MPa	Stress Amplitude Δ*σ*/MPa	Fatigue Life *N*_f_	Elongation
1	FAC-1	203.7	166.6	13,985	10.1%
2	FAC-2	203.7	166.6	16,870	6.0%

## Data Availability

The original contributions presented in this study are included in the article. Further inquiries can be directed to the corresponding author.
